# The characteristics and reproducibility of motor speech functional neuroimaging in healthy controls

**DOI:** 10.3389/fnhum.2024.1382102

**Published:** 2024-08-07

**Authors:** Katherine H. Kenyon, Frederique Boonstra, Gustavo Noffs, Angela T. Morgan, Adam P. Vogel, Scott Kolbe, Anneke Van Der Walt

**Affiliations:** ^1^Department of Neuroscience, School of Translational Medicine, Melbourne, VIC, Australia; ^2^Redenlab Inc., Melbourne, VIC, Australia; ^3^Murdoch Childrens Research Institute, Royal Children's Hospital, Melbourne, VIC, Australia; ^4^Department of Audiology and Speech Pathology, Faculty of Medicine, Dentistry and Health Sciences, Melbourne School of Health Sciences, University of Melbourne, Carlton, VIC, Australia; ^5^Department of Audiology and Speech Pathology, Parkville, VIC, Australia; ^6^Department of Neurology, Royal Melbourne Hospital, Melbourne, VIC, Australia

**Keywords:** speech production, reproducibility, fMRI, cerebellum, neurolinguistics

## Abstract

**Introduction:**

Functional magnetic resonance imaging (fMRI) can improve our understanding of neural processes subserving motor speech function. Yet its reproducibility remains unclear. This study aimed to evaluate the reproducibility of fMRI using a word repetition task across two time points.

**Methods:**

Imaging data from 14 healthy controls were analysed using a multi-level general linear model.

**Results:**

Significant activation was observed during the task in the right hemispheric cerebellar lobules IV-V, right putamen, and bilateral sensorimotor cortices. Activation between timepoints was found to be moderately reproducible across time in the cerebellum but not in other brain regions.

**Discussion:**

Preliminary findings highlight the involvement of the cerebellum and connected cerebral regions during a motor speech task. More work is needed to determine the degree of reproducibility of speech fMRI before this could be used as a reliable marker of changes in brain activity.

## 1 Introduction

Speech production includes the perceptual and motor components of language – how we produce the sounds used to share ideas and information (ASHA, 2020). It involves a complex combination of memory, perception, speech motor planning and execution. Even the final act of overt speech production is a complex, multisystem behaviour. Word selection and retrieval is primarily associated with the medial temporal and medial frontal gyri in the left cerebral hemisphere, and Crus I of the right cerebellar hemisphere (Price, 2012; Riès et al., 2017). Broca’s area plays a role in priming and preparing the motor system for articulation (Wildgruber et al., 1996; Watkins and Paus, 2004). This region of the left inferior frontal gyrus (IFG-L) coordinates information for the motor cortex prior to speech production (Flinker et al., 2015). The IFG-L is active during the word retrieval and response preparation stages of speech (Korzeniewska et al., 2011). Next, regions such as the premotor cortex, cerebellum, thalamus and putamen are involved in articulation alongside the lip, jaw and larynx regions of the primary motor and somatosensory cortices (Nota and Honda, 2004; Guenther, 2006; Price, 2012). The laryngeal musculature area of the primary motor cortex has also been linked to phonation and voluntary breath control required for speech (Correia et al., 2020). The onset of articulation occurs just prior to acoustic onset, or the vocalisation of speech (Mooshammer et al., 2012; Schaeffler et al., 2014; Jouen et al., 2021). Vocalisation similarly involves the bilateral primary motor and somatosensory cortices and inferior cerebellum, as well as the premotor cortex and supplementary motor area (Nota and Honda, 2004; Brown et al., 2021). While language is typically left lateralised, these motor components of speech production are more bilaterally represented within the cerebral cortex (Brown et al., 2009).

Studies using functional magnetic resonance imaging (fMRI) coupled with simple phrase (Nota and Honda, 2004), individual real word (Morgan et al., 2013), nonsense word (Pigdon et al., 2020) and syllable (Wildgruber et al., 2001; Riecker et al., 2005; Bohland and Guenther, 2006; Pützer et al., 2019) repetitions have assessed brain activity during speech production compared to either listening or resting baseline. Similar research investigating connectivity using fMRI and magnetoencephalography has highlighted the premotor cortex, orofacial regions of the motor cortex, medial and superior temporal cortices, cerebellum and IFG-L as key regions within the broader language production network (Kujala et al., 2006; Liljeström et al., 2015; Simonyan and Fuertinger, 2015). The orofacial motor cortex has been flagged as one of the most densely connected regions within the network, with the cerebellum also being mentioned as a highly connected area (Kujala et al., 2006; Simonyan and Fuertinger, 2015). Sensorimotor tasks activate the anterior cerebellum, while language-based tasks engage lobule VI and Crus I in the right cerebellar hemisphere (Stoodley and Schmahmann, 2009). Riecker et al. (2005) suggest the existence of two separate networks, one involved in motor preparation for speech and one for motor execution.

Speech is a known marker of neurological health, particularly in adult neurodegenerative conditions (Harris et al., 2019; Landin-Romero et al., 2021) but also increasingly seen as a promising marker of paediatric brain function (Morgan et al., 2016; Regier et al., 2016; Hiremath et al., 2021). Speech is a relatively straightforward and easy-to-collect behavioural output, making it a useful clinical marker across a range of conditions across the lifespan including autism spectrum disorders (Shriberg Lawrence et al., 2017; Talkar et al., 2020; Hiremath et al., 2021), mental health and psychotic disorders (Low et al., 2020; Koops et al., 2021). Speech has utility as a tool for differential diagnosis, and can be used to track change, as a response to treatment or disease progression (Morgan et al., 2013; Daudet et al., 2017; Chan et al., 2019; Noffs et al., 2020; Solomon Nancy et al., 2021).

To ensure robust cross-sectional individual participant or cohort findings and to enable longitudinal monitoring, it is crucial to understand the reproducibility of speech fMRI. There is currently no single definition of reproducibility used in scientific and clinical research. For this study, we use the definition of methods reproducibility outlined by Goodman et al. (2016): “the ability to implement, as exactly as possible, the experimental and computational procedures, with the same data and tools, to obtain the same results.” Typical group-level task-based fMRI analysis is generally considered to have a low test–retest reliability (Elliott et al., 2020; Noble et al., 2021). However, other research suggests that viewing all task-based fMRI as having poor reproducibility is overly simplistic and discounts studies showing higher reproducibility (Kragel et al., 2021). fMRI reproducibility appears to be highly variable and dependent on several factors, including the task, sample size, measures used, scanner noise and subject motion (Gorgolewski et al., 2013; Kragel et al., 2021). For example, a meta-analysis of fMRI task reproducibility reported intraclass correlation coefficients (ICCs) between 0.16 and 0.88 throughout the 13 included studies (Bennett and Miller, 2010). ICC looks at differences in brain activity between subjects or timepoints, and values range from −1 to 1. Scores <0 are indicative of no agreement between activation values at each timepoint and scores close to 1 show high agreement (Caceres et al., 2009; Nettekoven et al., 2018). Notably however, none of the studies in the meta-analysis involved speech. By contrast to these findings of non-linguistic tasks, language tasks including verb generation and sentence comprehension have shown high test–retest correlations, at least when assessing the lateralisation of language in the frontal and temporoparietal regions of the cerebral cortex, suggesting a high reproducibility of findings in this regard (Harrington et al., 2006). Further, Voyvodic (2012) used a sentence completion task to assess the reproducibility of language mapping fMRI in healthy participants. Using activation mapping as a percent of local excitation, the study found significant reproducible activation in the frontal and temporal regions (Voyvodic, 2012). Laterality and spatial extent of activation were found to have 95 and 55% similarity across time, respectively, (Voyvodic, 2012). Using both ICC and the Dice coefficient, Frankford et al. (2021) found high reproducibility of speech activation maps based on overt speech tasks using pseudoword and monosyllabic word repetition. The Dice coefficient describes the overlap of activation maps and is scored between 0 (no overlap) and 1 (complete overlap; Bennett and Miller, 2010; Frankford et al., 2021). Further, Nettekoven et al. (2018) found reliable activation during speech across time using a picture-naming task, focusing on IFG-L, Wernicke’s area (part of the left STG) and the primary motor cortex (Nettekoven et al., 2018). Similarly, a sentence completion task shows moderate reproducibility of activation in inferior frontal and temporo-parietal regions of the cerebrum (Elin et al., 2022). However, the mentioned studies have a cortical focus meaning other regions of the brain involved in motor speech production, including the cerebellum, were not highlighted in the research. Gorgolewski et al. (2013) found cerebellar activation in both a lip movement motor task and a word repetition task. The reproducibility findings from this study varied: the time-series correlations were low for both tasks, but moderate-to-high within-subject Dice overlap suggests higher reproducibility.

The present study used longitudinal fMRI (two timepoints, 6 weeks apart) to examine the brain networks involved in speech preparation and articulation during a word repetition task (Morgan et al., 2013) in healthy controls. We further aimed to determine the reproducibility of the fMRI signal during this task. The speech paradigm has previously been used in cross-sectional studies examining the neural structure of dysarthria following childhood brain injury and also in an inherited speech disorder, compared to healthy controls (Morgan et al., 2013; Liégeois et al., 2019), but not in healthy adults alone. Further, while a previous study included analysis on motor speech regions of the cerebral cortex (Frankford et al., 2021), it did not include non-cortical speech regions, nor were more complex words with more than two syllables presented in the speech tasks. This study is therefore novel in including whole brain analysis of motor speech related functional activation, and in using a speech paradigm with words of varying complexity, to assess motor speech fMRI reproducibility in healthy controls. We predicted that there would be significantly increased functional activation in the sensorimotor and speech regions of the cerebrum, as well as the putamen, thalamus, and cerebellum during speech production. Further, we hypothesised that, during speech preparation, we would see significant activation of IFG-L and the premotor cortex. We expected neural activation to remain relatively stable across the two time points, showing moderate reproducibility of the speech fMRI task.

## 2 Methods

### 2.1 Participants

Fifteen healthy volunteers aged 18–65 (45.4 ± 15.3y) participated in the study (75% female). These participants were right-handed and spoke English as their first language. The local Human Research Ethics Committee approved the study and all participants provided voluntary written consent. One participant was excluded from analysis due to significant head motion during scanning. Therefore, the final analysis included data from the remaining 14 participants.

### 2.2 Design and procedure

A longitudinal, repeated measures design with two timepoints 6 weeks apart (44 ± 5 days) was carried out with a sample of volunteers. At timepoint 1, each participant underwent several assessments, including taking of general medical history, detailed neurological assessment, and completion of the Scale of the Assessment and Rating of Ataxia to assess cerebellar function. Upper limb dystonia was also assessed using the Global Dystonia Scale. All participants scored zero on these scales. At both timepoints, participants underwent MRI scanning.

### 2.3 MRI acquisition

At timepoint 1, participants underwent a 3 T MRI scanning session (MAGNETOM TrioTim, Siemens, Medical Systems, Erlangen, Germany) to obtain: (a) T1-weighted volumetric sequence (TR = 11.0 ms, TE = 705.0 ms, FOV = 1536^*^1536 mm^2^, matrix = 208^*^256, slice thickness = 8.0 mm, flip angle = 7.0°), (b) Echo gradient echo planar imaging (EPI) fMRI (TR = 1.5 ms, TE = 33 ms, FOV = 204^*^204 mm, matrix = 104^*^104, voxel size = 2.0 × 2.0 × 2.0 mm, slice thickness = 2 mm, flip angle = 85.0°, volumes = 200, GRAPPA acceleration factor of 2, multi-band slice acceleration factor of 3). Each EPI run involved administering the audio speech task once over 6 min, providing us with two EPI runs of the speech task at timepoint 1. At timepoint 2, participants had another fMRI run with the same settings. Using an MRI-compatible microphone, audio recordings of the speech task responses were acquired for each participant. The response timing was then used to identify functional activation during only the motor speech production aspect of the task at each timepoint.

### 2.4 Audio speech task

**Figure 1:**
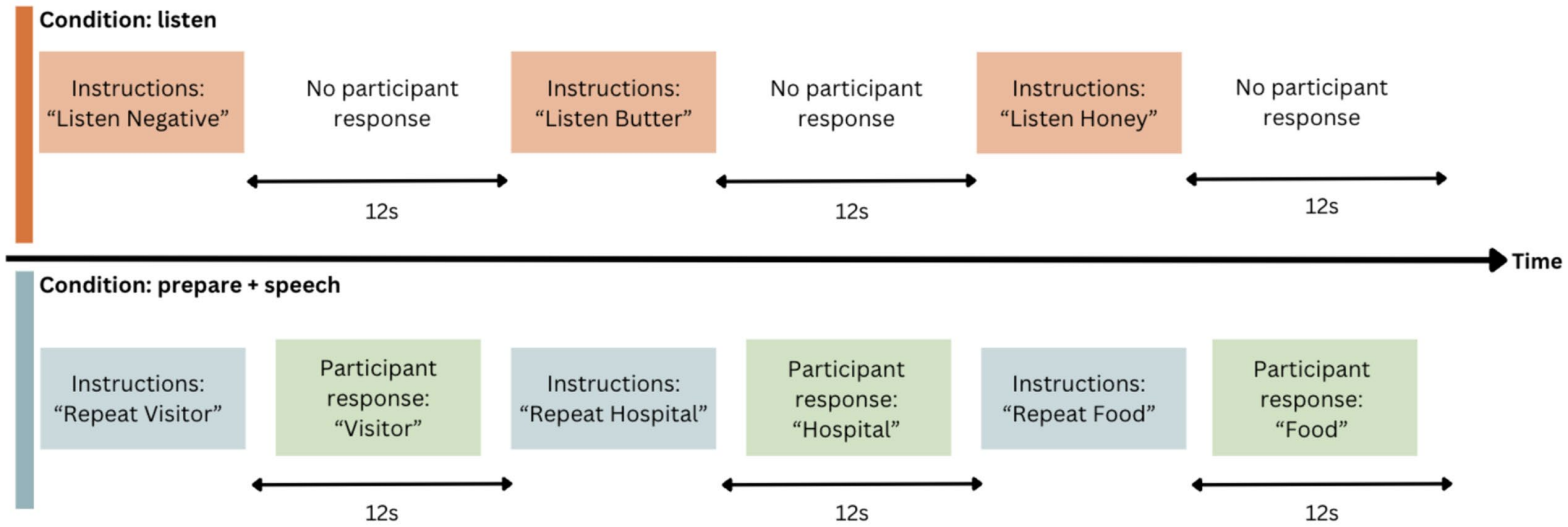
Event-related design for speech task. Word order and condition are pseudorandomised.

Imaging data were collected while participants completed a six-minute speech production task with an event-related design. Participants listened to an audio recording that presented 30 single words, followed by the instruction to either “listen” (“listen” condition) or “repeat” (“prepare + speech” condition) the word, at 12 s intervals (see Figure 1). The 12 s period for each stimulus was designed to include 2.5 s for stimulus presentation, 2.0 s for participant response, and 7.5 s to capture peak haemodynamic response (Morgan et al., 2013). The speech task was repeated at each timepoint, with two EPI runs conducted each time as noted above. This provided us with two 12-min sets of data, each with 60 words. At both MRI data acquisition timepoints, both word and instruction order were pseudorandomised to improve internal consistency and minimise practice effects (Morgan et al., 2013; Appendix 1).

### 2.5 Imaging analysis

**Figure 2:**
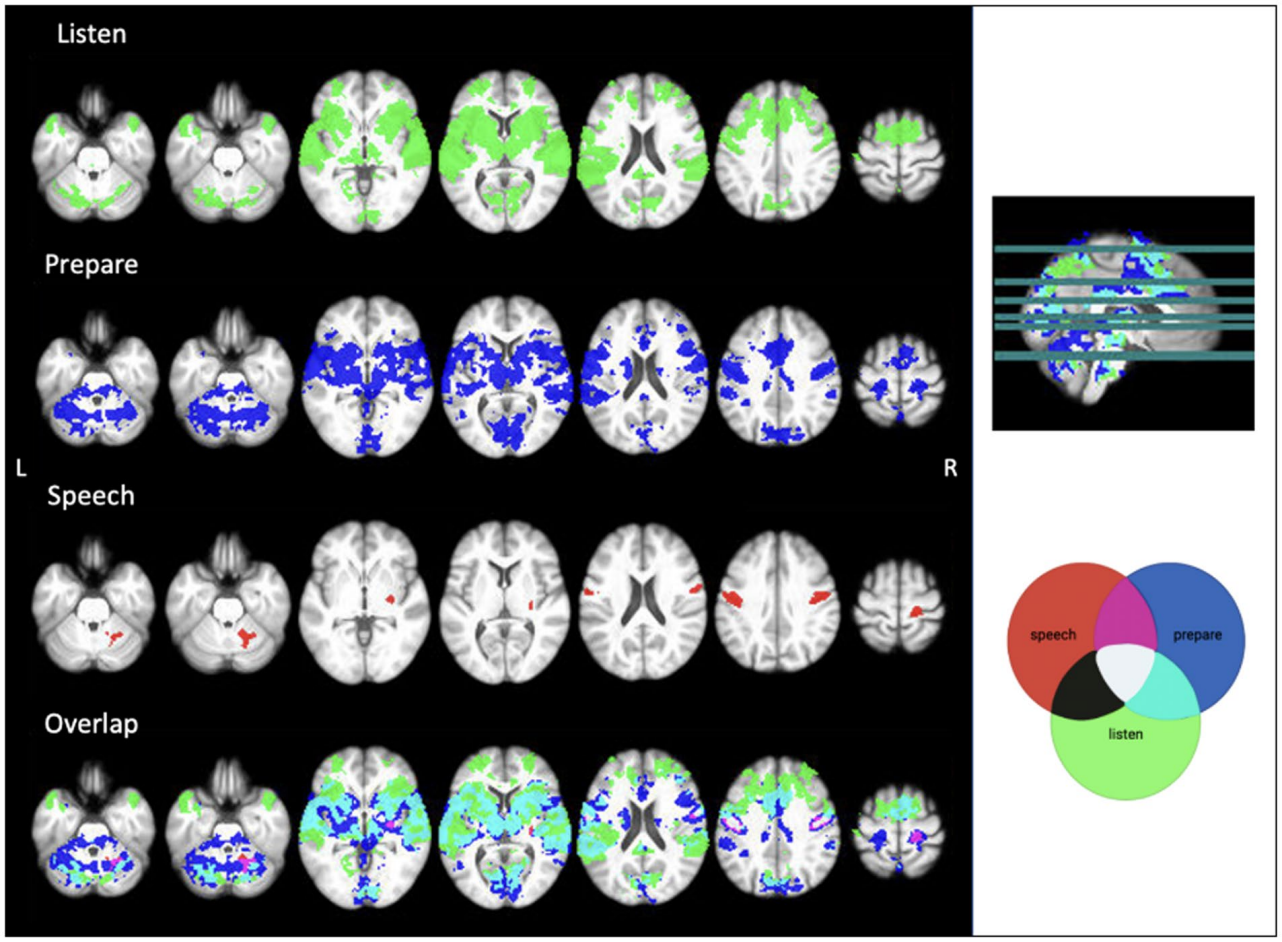
Average functional activation during each aspect of the audio speech task. Image created using MRIcron. Threshold z = 3.1.

To determine speech task timing for analysis, we separated the two conditions of the task into three aspects: (1) “listen” when the participant listens to the word without having to repeat it, (2) “prepare” when the participant listens to the word that they must subsequently repeat, and (3) “speech” when the participant says the word out loud. “Listen” and “prepare” timing was set for each run of the speech task, and “speech” timing was determined manually from audio recordings in the scanner for each run for each participant. This timing was used to establish functional activity during the 1.5 s following the onset of either the task instruction (“listen” or “prepare”) or active speech production. We used FEAT v6.00 (FSL, FMRIB, Oxford, UK) to perform fMRI analyses. Raw fMRI scans were pre-processed to correct for head motion [MCFLIRT motion correction (Jenkinson et al., 2002)], spatially smoothed (4 mm extent threshold) and registered to the main structural image using boundary-based linear registration. The scans were also registered to standard MNI space using FNIRT nonlinear registration. Performing speech tasks during image acquisition can impact the activation signals of interest (Gracco, Tremblay & Pike, 2005). Given the nature of the speech task, we then used ICA AROMA to further correct for head movement (Pruim et al., 2015). The combination of MCFLIRT and ICA AROMA has previously been used to correct for speech-related motion artifacts (Janssen and Mendieta, 2020). At this point, one participant was excluded due to significant motion remaining after both standard and additional motion correction. We used multi-level general linear model (GLM) analysis to identify regions of significant activation. We combined the two runs at each timepoint then ran analyses separately for each participant at timepoint 1 (TIME1) and timepoint 2 (TIME2). This level also calculated the main effects and calculated the level and location of functional activation at the listen, prepare and speech timestamps over both TIME1 and TIME2. Results from this level were used for single subject reproducibility analysis. Higher-level analysis was then used to determine common areas of brain activation across participants. In this level, we assessed the activation at each timepoint separately, and combined all data to find the group average activation maps for each aspect of the speech task at both timepoints. We used a z-stat threshold of >3.1 to identify regions of significant activation during “listen,” “prepare,” and “speech.” This threshold was also used to correct cluster size familywise error at *p* < 0.05. Given this was an exploratory study, we used the average regions of activation during “speech” as regions of interest (ROI, see Figure 2 “speech” activation map). Using the *Harvard-Oxford cortical and subcortical atlases* for labelling purposes, we found activation cluster 1 in the right putamen, and clusters 2, 4 and 5 in the sensorimotor areas of the pre-and post-central gyri, primarily in orofacial regions. The *cerebellar atlas in MNI152 space after normalisation with FNIRT* was used to identify the significant cerebellar activation during speech in the right anterior cerebellar lobules IV and V (Diedrichsen et al., 2009). We then compared the magnitude of activation within these clusters during speech production across TIME1 and TIME2.

### 2.6 Statistical analysis

To assess reproducibility, we compared speech-related functional activation within each average ROI at TIME1 with that at TIME2 (6 weeks later). We first used paired t-tests (*p* < 0.05) to determine whether there were any significant differences in activation between the timepoints. Reproducibility was then quantified using ICC [based on the 95^th^ percentile, absolute agreement, 2-way mixed-effects model (Barry et al., 2016; Koo and Li, 2016)], and the average coefficient of variation (CoV). The 95th percentile was used for these analyses due to its equivalence with a significance level of *p* = 0.05 and to protect against plausible but false high single-voxel correlations (Barry et al., 2016, 2018; Paul et al., 2017; Turner et al., 2018).

#### 2.6.1 Within-subject reproducibility

For single subject ICCs, we created four masks – bilateral IFG and STG, subcortical (thalamus and putamen), and cerebellum, and compared the z-stat at the 95^th^ percentile within each mask for each participant. For the CoV, we first calculated the means and standard deviations for each ROI between the two timepoints for each participant. Dividing the standard deviations by the means gave us the CoV for each ROI in each participant.

#### 2.6.2 Group-level reproducibility

For the group ICC, we compared the z-stat at the 95^th^ percentile for eachROI between TIME1 and TIME2. The group-level CoVs for each ROI was the average CoV across participants. We then assessed activation location similarity within the ROIs identified with the GLM between timepoints and quantified this using a Dice coefficient.

## 3 Results

### 3.1 Listen/prepare/speech contrasts

Our GLM analysed brain activity during the listen, prepare, and speech aspects of the task. Results show widespread activation during “listen” and “prepare,” with large amounts of overlap in activated areas, including the cerebellum and sensory, motor, and auditory regions of the cerebral cortex. Additionally, there was significant overlap in activated regions between “prepare” and “speech,” largely shown in the cerebellum and bilateral sensorimotor cortices. See Figure 2 for activation patterns.

### 3.2 Validation of the audio speech task

**Figure 3:**
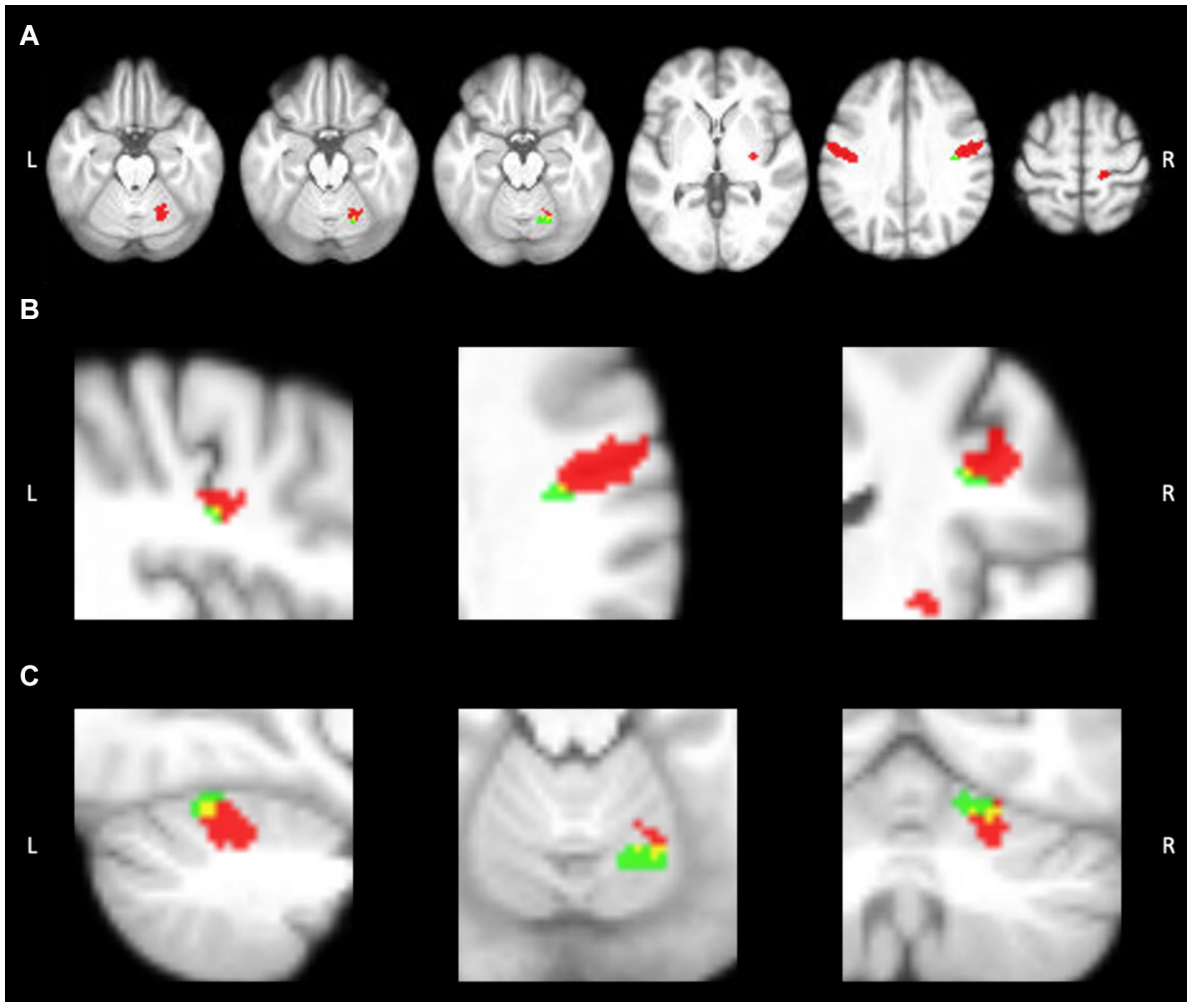
Activation within ROIs at timepoint 1 (red) and timepoint 2 (green). Overlap can be seen in yellow. **(A)** Axial view of functional activation during speech production. **(B)** Activation overlap within the R sensorimotor cortex ROI. **(C)** Activation overlap within the R anterior cerebellar ROI. Image created using MRIcron.

While we looked at all three aspects of the task, we focused on “speech,” given it is the aspect specifically highlighting motor speech production. No significant differences were found between TIME1 and TIME2 for any aspect of the speech task. During “speech”, we identified five average regions of significant activation (*z*-score > 3.1) The ROIs for this analysis identified using the GLM included bilateral sensorimotor cortices, right putamen, and right anterior cerebellum. We additionally found location overlap between TIME1 and TIME2 in the right sensorimotor cortex and right anterior cerebellar ROIs. The Dice similarity coefficients were 0.005 and 0.085, respectively. All functional activation in the right sensorimotor cortex at TIME1 and TIME2 was within the right Brodmann area 3a. In the right anterior cerebellum, 96% of activated voxels at TIME1 and 86% of activated voxels at TIME2 were within the right lobule V. See Figures 2, 3 and Tables 1, 2 for further detail.

**Table 1:** Overlap of activation location during “speech” between timepoint 1 and timepoint 2.

ROI	Anatomical region	Timepoint	Voxels	MNI coordinates (mm)			Dice coefficient
				x	y	z	
3	Anterior cerebellum, R	1	108	20	−56	−26	0.085
		2	43	16	−64	−16	
5	Sensorimotor cortex, R	1	383	42	−8	34	0.005
		2	13	38	−16	32	

R, right hemisphere; L, left hemisphere.

**Table 2:** Average regions of interest during “speech” and average activation of cluster coordinates in MNI space at peak activation.

ROI	Voxels	Anatomical region	MNI coordinates (mm)			*Z*-score (max)	ICC	CoV	*t*-stat
			x	y	z				
1	78	Putamen, R	30	−16	−2	4.1	−0.397	1.533	0.547
2	92	Upper sensorimotor cortex, R	20	−28	66	4.35	−0.255	2.927	1.521
3	287	Anterior cerebellum, R	16	−64	−16	4.3	0.499^*^	0.304^*^	1.305
4	493	Sensorimotor cortex, L	−54	−12	34	4.76	0.066	1.054	1.524
5	507	Sensorimotor cortex, R	50	−6	32	4.58	0.002	7.501	1.268

R, right hemisphere; L, left hemisphere.

* *p* < 0.05.

### 3.3 Reproducibility

**Figure 4:**
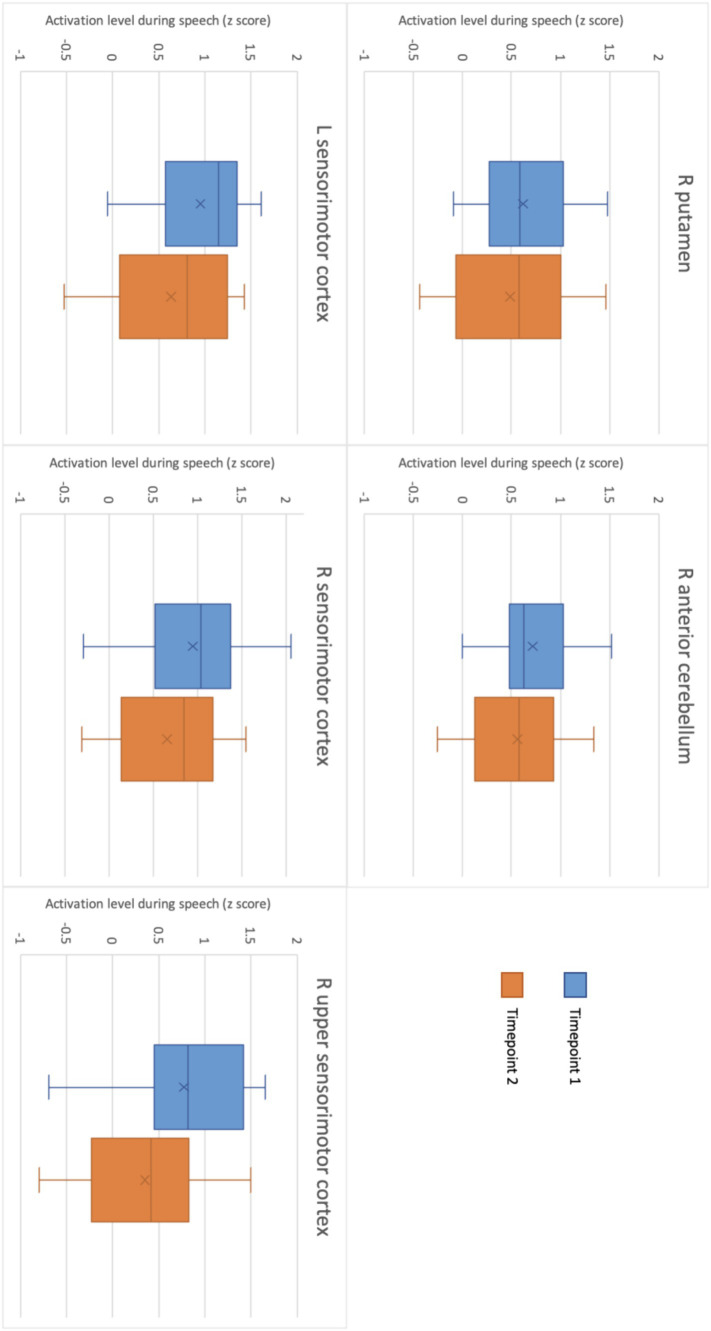
Level of activation during motor speech production in each ROI at timepoint 1 and at timepoint 2.

Reproducibility was determined using ICC and CoV between TIME1 and TIME2. As determined by the *t*-test and comparison of functional activation within the GLM, there were no significant group differences in activation magnitude found between the two timepoints (see Table 2; Figure 4). Individual ICCs varied widely (see Table 3). The cerebellar ICCs were found to be significant (*p* = 0.038). Cortical and subcortical ICCs were not significant. Group ICC ranged from 0.002 to 0.499. Only the cerebellar activation cluster in the right lobules IV and V had a significant moderate correlation between TIME1 and TIME2, with an ICC of 0.499 (*p* = 0.003) and CoV of 30.4%. ICCs for activation within the other ROIs did not approach significance.

**Table 3:** Individual intraclass correlations for cerebellar, cortical, and subcortical speech regions.

Subject	Cerebellum (μ, sd)	IFG (μ, sd)	STG (μ, sd)	Subcortical (μ, sd)
1	0.450 (0.045, 0.676)	0.592 (0.118, 0.681)	0.398 (0.215, 0.762)	0.987 (0.153, 0.663)
2	0.334 (0.655, 0.662)	0.279 (0.464, 0.668)	0.353 (0.676, 0.736)	0.571 (0.701, 0.565)
3	0.555 (0.479, 0.612)	0.682 (0.289, 0.754)	0.669 (0.463, 0.627)	0.952 (0.373, 0.629)
4	0.708 (0.425, 0.669)	0.898 (0.209, 0.549)	0.993 (0.662, 0.754)	0.906 (0.316, 0.704)
5	0.553 (0.325, 0.731)	0.519 (0.064, 0.786)	0.668 (0.604, 0.767)	0.669 (0.136, 0.681)
6	0.964 (0.395, 0.702)	0.806 (0.124, 0.729)	0.931 (0.670, 0.734)	0.783 (0.158, 0.701)
7	0.838 (0.087, 0.579)	0.876 (0.112, 0.588)	0.936 (0.389, 0.530)	0.743 (0.079, 0.620)
8	0.999 (0.102, 0.595)	0.644 (−0.032, 0.592)	0.777 (0.099, 0.487)	0.811 (−0.098, 0.589)
9	0.892 (0.291, 0.716)	0.388 (0.019, 0.613)	0.882 (0.472, 0.626)	0.705 (0.266, 0.642)
10	0.867 (−0.079, 0.596)	0.556 (0.440, 0.683)	0.684 (0.191, 0.642)	0.112 (0.085, 0.615)
11	0.402 (0.054, 0.619)	0.579 (0.036, 0.635)	0.484 (0.203, 0.617)	0.776 (0.067, 0.574)
12	0.820 (0.168, 0.663)	0.546 (0.066, 0.555)	0.335 (0.317, 0.576)	0.202 (0.185, 0.665)
13	0.880 (−0.234, 0.622)	0.366 (−0.085, 0.564)	0.726 (−0.107, 0.568)	0.997 (0.283, 0.614)
14	0.873 (0.148, 0.617)	0.402 (0.283, 0.629)	0.988 (0.281, 0.635)	0.389 (0.319, 0.616)

ICC, intraclass correlation; IFG, inferior frontal gyrus; STG, superior temporal gyrus.

## 4 Discussion

This study examined functional characteristics of speech during a speech fMRI task in healthy controls and determined its reproducibility. Speech-related activation was observed in the right cerebellar lobules IV and V, and connected regions of the cerebrum involved in speech, including the right putamen and bilateral sensorimotor regions. These findings support our first hypothesis and earlier work describing cerebellar contributions to speech production. The right cerebellum is thought to play a key role in motor speech production, as there is greater contralateral connectivity between the motor cortex and the cerebellum than there is bilateral connectivity (Krienen and Buckner, 2009). This implies that during speech, where the left motor cortex is active, the right cerebellum should show higher levels of activity. While there is bilateral cerebellar activation during articulation, it is higher in both magnitude and extent in the right superior cerebellum (Ackermann et al., 1998; Chen and Desmond, 2005). Crus I and cerebellar lobule VI exhibit load-dependent activation with IFG-L during articulatory rehearsal tasks, with larger areas of activation in the right cerebellar hemisphere (Chen and Desmond, 2005). This explains our findings regarding activation in the right cerebellum during “speech” and the motor cortices during both “prepare” and “speech.”

In terms of the reproducibility of the task, we saw no significant group differences in functional activation between timepoints. Group level right anterior cerebellar activity during speech was somewhat consistent between the two timepoints, with a moderate ICC of 0.499. Individual subject ICCs varied widely, with significant cerebellar ICCs showing low to high reproducibility depending on the participant. No other individual ICCs were significant. These findings suggest reasonable reproducibility of cerebellar activation during this specific speech fMRI task at both an individual and group level. By contrast however, no cerebral activated regions at TIME1 showed a significant voxel-wise correlation with activity in the same regions at TIME2, either in individual or group level analysis. The Dice coefficients of overlapping regions of activation within these were also very low, indicating minimal thresholded activation pattern overlap. This is a critical finding and surprising given the higher level of reproducibility of cerebral activation seen to date in this field, even when using similar tasks (Voyvodic, 2012; Nettekoven et al., 2018; Elin et al., 2022). We note here that we used the z-score at the 95^th^ percentile for ICC, due to its equivalence to a significance level of *p* = 0.05 (Barry et al., 2016; Paul et al., 2017; Turner et al., 2018). We do, however, acknowledge that there are other ways to investigate this, including taking the median or maximum contrast value, the contrast at peak activation, or the ICC between measurements (Raemaekers et al., 2007; Caceres et al., 2009). This variation in voxel-wise reproducibility between ROIs may be due to individual psychophysiological factors such as fatigue and attention, variance in noise in the fMRI signal, activation outliers, thresholding sensitivity, or practice effects and changes in task strategies between timepoints (Machielsen et al., 2000; Yoo et al., 2005). We must also address the potential influence of practice effects on functional activity at TIME2. Previous research using a picture naming task found a decrease in BOLD signal at the time of the second scan compared to the first (Basso et al., 2013). Though the difference in activation between timepoints was non-significant, this is what we observed (Figure 4). As an exploratory study, the ROIs used for this analysis were also based on average level of activation during speech in this cohort, and thus were quite small. This would also impact reproducibility. While we only see minor voxel-wise overlap in activation patterns between timepoints, we do see very close activation in both the right sensorimotor and right cerebellar ROIs. All functional activation within the right sensorimotor cortex at both timepoints was confined to Brodmann area 3a, providing evidence for higher levels of reproducibility than was captured using voxel-wise overlap analyses. Given the potential influence of head motion and position in the scanner on fMRI signal (Power et al., 2014), and the exploratory nature of this study, we suggest further research be conducted using larger ROIs chosen based on past findings to improve identification of motor-speech related functional activation and increase the accuracy of reproducibility measures. It may also be beneficial to use a sparse sampling design rather than an event-related design for the speech fMRI task. In saying that, Yoo et al. (2005) found that the cerebellum had the greatest activation volume overlap between intrasession scans during a motor task when compared to activation in the motor and supplementary cortices. Further, when looking at intersession scans, activation in both the cerebellum and motor regions of the cerebrum were reproducible (Yoo et al., 2005).

As regards to the three specific “listen,” “prepare,” and “speech” components of the task, temporal and frontal regions were activated during the “listen” and “prepare” segments. Temporal and frontal regions are involved in auditory processing (Schirmer et al., 2012; Lima et al., 2016) and speech comprehension (Yue et al., 2013), and are connected to the cerebellum (Buckner et al., 2011). Results also show a significant overlap in areas of activation between “prepare” and “speech,” particularly in the cerebellum and sensorimotor cortices in the cerebrum. This observation provides possible evidence of a priming effect in these regions before speech articulation. Moreover, we see activation of IFG-L during “prepare,” but not during “speech,” which suggests a priming effect during “prepare” and explains why we do not see significant IFG-L activation during “speech.” This is consistent with previous research on the role of IFG-L in speech preparation (Wildgruber et al., 1996; Watkins and Paus, 2004; Korzeniewska et al., 2011; Flinker et al., 2015).

## 5 Limitations

The study had a small sample size. Recent research demonstrates that sample size can have a considerable impact on the reproducibility of task-based fMRI (Turner et al., 2018; Bossier et al., 2020), including a high false negative rate (Lohmann et al., 2017). As such, there were few findings that were reproducible at timepoint 2. This lack of reproducibility could potentially be addressed by repeating this experiment with a larger group of participants. We acknowledge that our small sample also has a wide age range and are aware of the potential effect of age on functional activity. A recent meta-analysis indicates altered functional activation related to motor control in older adults (Zapparoli et al., 2022). Moreover, Tremblay et al. (2017) suggest that changes in motor and executive control contribute to age differences in brain function associated with speech production. This age effect additionally may have impacted reproducibility through inter-subject variability. We also did not correct for multiple comparisons. As the study called for planned comparisons, we directly compared activation within each of our ROIs between timepoints individually. We were able to control for some variables including keeping the method and environment stable, yet other aspects of fMRI that may influence reproducibility are less modifiable. Potential variables include differences in heart rate or respiration between participants affecting the BOLD signal, and physiological noise such as underlying brain activity. Further, imaging artifacts such as machine noise and head movement are more pronounced during a speech task. While the analysis included standard motion correction and the use of ICA AROMA, more substantial head movement cannot be controlled for. This led to the exclusion of one participant, further decreasing sample size. There is also the possibility of behavioural differences between sessions impacting functional activation. Due to the words presented being the same at both timepoints and the sample consisting only of healthy controls, we did not take this into account for our study. However, for further research, particularly in clinical populations, we suggest adding behavioural data as a variable. The GLM and t-tests found no significant differences in functional activation between timepoints, thus implying high reproducibility. In contrast, the ICC appears to be much more sensitive to the thresholding, picking up smaller variations in voxel-to-voxel activation than the t-tests, leading to lower correlation between timepoints and thus lower implied reproducibility of the findings. Similarly, Dice coefficients are sensitive to both thresholding level and area of calculation, where lower threshold values and whole-brain analysis increase the overlap (Frankford et al., 2021). We used standard thresholding throughout this study, but only calculated Dice coefficients within ROIs, likely decreasing the level of overlap found and lowering the calculated reproducibility. Finally, the speech task used required the articulation of single words in a list and a natural speech sample may improve ecological validity.

### 5.1 Concluding statements

Our findings further confirm that a simple fMRI speech task can allow observation of brain activity associated with motor speech preparation and production. We found significant activation in the right putamen, right anterior cerebellum, and bilateral sensorimotor cortices during speech production, in line with past work. Additionally, widespread activation through bilateral cerebellar, sensorimotor, premotor, and temporal regions was observed during “prepare,” consistent with speech perception and motor speech preparation.

Initial analyses reflected no significant differences in functional activity during speech production between our initial assessment and the second session after 6 weeks, which is suggestive of good reproducibility. Most critically, further statistical analyses showed only moderate reproducibility in cerebellar activation associated with motor speech production, and did not highlight other regions of activation as reproducible. This discrepancy is likely due to varying sensitivity to the thresholds used, but does emphasise the significant and consistent role of the cerebellum in motor speech activity. We suggest future research further investigate the potential of monitoring speech-related cerebellar activity specifically. Injury to the cerebellum can significantly impair speech, leading to a decline in quality of life (Walshe and Miller, 2011; Noffs et al., 2020). This is seen in people with neurodegenerative diseases (Piacentini et al., 2014; Lirani-Silva et al., 2015; Leite and Constantini, 2017; Noffs et al., 2018), stroke survivors (Spencer and Brown, 2018) and cases of traumatic brain injury (Gandhi et al., 2020). Further assessing this speech task’s psychometric properties could lead to its use for diagnosis of such conditions.

We can conclude that the word repetition task can assess activity in the cerebellum during motor speech preparation and production. While this is promising, further research is required to determine whether the task could be used to monitor any changes in cerebellar speech activity over time. Further, future research ought to investigate whether this task could be used as a speech marker of disease progression in diseases with significant cerebellar impact, such as multiple sclerosis or Parkinson’s disease (Noffs et al., 2018, 2020; Magee et al., 2019; Rusz et al., 2019).

## Data availability statement

The data analyzed in this study is subject to the following licenses/restrictions: data is available upon request. Requests to access these datasets should be directed to AnV, anneke.vanderwalt@monash.edu.

## Ethics statement

The studies involving humans were approved by Monash University Human Research Ethics Committee. The studies were conducted in accordance with the local legislation and institutional requirements. The participants provided their written informed consent to participate in this study.

## Author contributions

KK: Conceptualization, Formal analysis, Project administration, Validation, Visualization, Writing – original draft, Writing – review & editing. FB: Conceptualization, Data curation, Investigation, Resources, Supervision, Writing – review & editing. GN: Data curation, Formal analysis, Supervision, Writing – review & editing. AM: Methodology, Resources, Writing – review & editing. AdV: Software, Supervision, Writing – review & editing. SK: Conceptualization, Methodology, Resources, Supervision, Validation, Writing – review & editing. AnV: Conceptualization, Funding acquisition, Project administration, Resources, Supervision, Writing – review & editing.
