# Supplementary material for: The characteristics and reproducibility of motor speech functional neuroimaging in healthy controls
Source: Front Hum Neurosci. 2024 Aug 7;18:1382102. doi: 10.3389/fnhum.2024.1382102 (PMC11335534; doi:10.3389/fnhum.2024.1382102)
Supplement: Supplementary file 1 [file Table_1.DOCX]

**Supplementary table 1**. Speech task word list

| **ITEM** | **R/L** | **WORD** |
| --- | --- | --- |
| 1 | Repeat | Calendar |
| 2 | Listen | Yesterday |
| 3 | Listen | Cab |
| 4 | Repeat | Butter |
| 5 | Listen | Magic |
| 6 | Repeat | Honey |
| 7 | Repeat | Negative |
| 8 | Listen | People |
| 9 | Repeat | Fantastic |
| 10 | Repeat | Neck |
| 11 | Repeat | Tail |
| 12 | Listen | Charity |
| 13 | Repeat | People |
| 14 | Repeat | Justify |
| 15 | Repeat | Mess |
| 16 | Listen | Tail |
| 17 | Listen | Neck |
| 18 | Listen | Fantastic |
| 19 | Repeat | Charity |
| 20 | Repeat | Magic |
| 21 | Repeat | Cab |
| 22 | Listen | Mess |
| 23 | Repeat | Author |
| 24 | Listen | Negative |
| 25 | Listen | Butter |
| 26 | Listen | Honey |
| 27 | Listen | Justify |
| 28 | Repeat | Yesterday |
| 29 | Listen | Author |
| 30 | Listen | Calendar |
| 31 | Listen | Food |
| 32 | Repeat | Logical |
| 33 | Listen | Coin |
| 34 | Listen | Threatening |
| 35 | Repeat | Crash |
| 36 | Listen | Crash |
| 37 | Listen | Broken |
| 38 | Repeat | Kitchen |
| 39 | Repeat | Zero |
| 40 | Listen | Hospital |
| 41 | Repeat | Fruit |
| 42 | Listen | Fruit |
| 43 | Listen | Glory |
| 44 | Repeat | Dish |
| 45 | Repeat | Finger |
| 46 | Repeat | Threatening |
| 47 | Listen | Kitchen |
| 48 | Repeat | Visitor |
| 49 | Repeat | Hospital |
| 50 | Repeat | Food |
| 51 | Listen | Visitor |
| 52 | Listen | Logical |
| 53 | Listen | Dish |
| 54 | Repeat | Broken |
| 55 | Listen | Gab |
| 56 | Repeat | Glory |
| 57 | Repeat | Coin |
| 58 | Repeat | Gab |
| 59 | Listen | Finger |
| 60 | Listen | Zero |
